# Anterior anal sphincter repair can be of long term benefit: a 12-year case cohort from a single surgeon

**DOI:** 10.1186/1471-2482-7-1

**Published:** 2007-01-11

**Authors:** Benjamin R Grey, Rowena R Sheldon, Karen J Telford, Edward S Kiff

**Affiliations:** 1 Department of General Surgery, South Manchester University Hospitals NHS Trust, Manchester, UK

## Abstract

**Background:**

Early surgical results of anterior sphincter repair for faecal incontinence can be good, but in the longer term are often disappointing. This study aimed to determine the short and long term outcomes from anterior sphincter repair and identify factors predictive of long term success.

**Methods:**

Patients who underwent anterior sphincter repair between 1989 and 2001 in one institution were identified. Postal questionnaires were sent to patients, which included validated scoring systems for symptom severity and quality of life assessments for faecal incontinence. Patient demographics and risk factors were recorded as were the results of anorectal physiology studies and endoanal ultrasound.

**Results:**

Eighty-five patients underwent repair by one consultant. The length of follow up ranged from 1 to 12 years. Most patients (96%) had early symptom improvement postoperatively. Of the 47 patients assessed long term (≥ 5 years), 28 (60%) maintained this success. Significant improvements in quality of life were observed (P < 0.001). Neither patient, surgical nor anorectal physiology study parameters were predictive of outcome.

**Conclusion:**

There were no predictive factors of outcome success and no changes in anal manometry identified, however anterior sphincter repair remains worthwhile. Changes in compliance of the anorectum may be responsible for symptom improvement.

## Background

Faecal incontinence can result from disruption to the anal sphincter muscles; this can be due to traumatic, surgical or obstetric causes [1]. Anterior sphincter repair (ASR), or sphincteroplasty, remains a common surgical procedure for treatment of faecal incontinence in the presence of a defect in the external anal sphincter (EAS) muscle. The results of ASR are often good in the early post operative period, but in the longer term results are more disappointing [2].

The majority of patients undergoing ASR are female, and have incurred obstetric injury to the sphincters at the time of vaginal delivery [3]. Delayed repair is frequently performed because of unrecognized injury at the time of childbirth or failure of primary repair. A number of studies have looked into the functional outcome following overlapping sphincteroplasty with most studies reporting an early success rate between 70 and 90% [4,5].

It is known that neuropathic damage to the anal sphincter can occur in association with obstetric disruption and that faecal incontinence in these cases is often more insidious in its onset [6]. Pudendal neuropathy has been reported as a predictor of failure following sphincteroplasty [7,8]. Other studies have failed to identify any relationship and concluded that repair of anatomical sphincter defects should still be considered in the presence of pudendal neuropathy [9,10]. Most studies reported a good short term outcome of sphincteroplasty and that success rates appeared lower in studies that had a longer period of follow up. Recently Malouf et al [11] and Halverson and Hull [2] published results of sphincteroplasty at five years following surgery and found that only half of patients were continent to stool.

Previous studies have looked for predictive measures of successful outcome following ASR, but no consistent factors have been identified [2,12]. Comparison of previous studies is difficult as there is no uniform method of assessment for postoperative outcome or success.

The aims of this study were to determine short and long term outcomes in patients requiring ASR using validated scoring systems and identify any factors predictive of successful long term outcome.

## Methods

All patients that had undergone ASR at South Manchester University Hospitals NHS Trust were identified from hospital records. The selection of patients with faecal incontinence for ASR was based on the history of a potential mechanism of injury and examination findings of a defect in the EAS. Sphincter defects were identified by digital examination or by endoanal ultrasound, which was introduced to the department in 1996 as part of routine preoperative assessment.

### Operative technique

Patients underwent full bowel preparation with Sodium Picosulphate and received thromboembolic prophylaxis (thromboembolic deterrent stockings and low molecular weight heparin) the day prior to surgery. A single dose of Cefuroxime and Metronidazole was given at induction of anaesthesia. The patients were placed in the lithotomy position. A transverse curvilinear incision was then made in the perineum and the vaginal wall dissected to the recto-vaginal septum. This dissection was then continued following the rectal border laterally to identify the levator ani muscles on either side. The anal mucosa was dissected from the remains of the anal canal musculature to the level of the pelvic floor. The intersphincteric plane was dissected enabling repair of the internal anal sphincter if required. A levatoroplasty was then performed with the aim being to lengthen the anal canal by effectively carrying out a posterior colporrhaphy. Interrupted absorbable sutures were used to draw together the fascia over pubovaginalis from either side over a length of some 5 cm. The repair continued to the external anal sphincter where an overlapping sphincteroplasty was performed. The wound was closed longitudinally leaving a small gap for drainage. A urinary catheter was placed at the end of the procedure and removed within 48 hours.

### Clinical result

The clinical outcomes were determined from hospital records and postal questionnaires sent to all patients at the time of the study. Hospital notes were reviewed to obtain demographic details, co-morbidity, clinical findings, operative procedure and postoperative details. Preoperative anal manometry, endoanal ultrasound, pudendal nerve terminal motor latency (PNTML) and rectal sensitivity parameters were also recorded. Anal manometry measured maximal resting pressure (MRP), maximal voluntary contraction (MVC) and maximal squeeze pressure (MSP). MSP was calculated by subtracting MRP from MVC. The postal questionnaire was divided into two sections, one recording symptom severity and quality of life scores prior to surgery, the other section after ASR. Non-responders were sent a further set of questionnaires at the postal address known to the hospital. Long term outcomes were determined after 5 years of follow up.

### Questionnaires

Each section (pre- and post-operative) of the questionnaire was colour co-ordinated to aid interpretation and each section had three elements.

1. The St. Mark's scoring system [13] assessed the degree of faecal incontinence based on the type of incontinence (flatus, liquid and solid) and the use of constipating agents and pads. It consists of seven questions and is scored from of zero to 24, zero representing total continence and 24 total incontinence.

2. The Manchester Health Questionnaire [14] is a validated quality of life questionnaire specific for faecal incontinence. It covers aspects of lifestyle including general health, incontinence impact, physical limitations, social limitations, emotions and personal relationships. It is scored over 31 questions each with five answer options ranging from never to always. The values are then converted into a percentage score.

3. Also included was a numerical linear patient assessment score of faecal incontinence in which patients were asked to mark on a scale of one to 10 (one totally incontinent, 10 total continence) their perceived level of continence both before and after surgery.

Two additional questions were asked in the postoperative section of the questionnaire. Patients were asked to select one of four categories relating to symptom improvement after surgery – improved, initially improved then deteriorated, same or worse. The second question asked was: "Would they recommend the operation to a friend with faecal incontinence?"

Postoperative outcome scores were compared with preoperative physiological parameters. Endoanal ultrasound was included as part of preoperative assessment from 1996; these results were compared from that time.

### Statistical analysis

Questionnaire responses were analysed using the paired T test, Wilcoxon Signed Rank test, and Mann-Whitney U test. Categorical data was analysed using the Chi Squared test. Results were considered significant at the p ≤ 0.05 level.

## Results

Over a 12 year period (1989–2001) 85 anterior sphincter repairs were carried out by one consultant surgeon. Eighty-two patients were female. The median age at the time of surgery was 46 years (range 22–80 years). The duration of symptoms at the time of surgery was a median of four years (range 1–40 years).

Any potential mechanism of sphincter injury was recorded from hospital notes. Of the 82 women in this group an obstetric cause (tear or episiotomy) was recorded in 59 (72%) cases. Other possible mechanisms recorded were; abscess or fistula; haemorrhoid or fissure surgery; imperforate anus reconstruction. Six (7%) patients had more than one mechanism recorded, and 18 (22%) patients had no recorded mechanism in the notes. Thirty-two (39%) patients had previously undergone surgical repair of the anal canal or pelvic floor.

Anorectal physiology studies were recorded in 79 (91%) of patients. A normal PNTML (≤ 2.2 ms) was measured in 48 patients (61%), 17 (22%) had a unilateral neuropathy and nine (11%) had bilateral neuropathy. Five (6%) patients had no recorded values due to either pain or no elicited response.

The length of stay postoperatively was a median of seven days (range 2–33 days). There was no reported post-operative mortality. Morbidity was recorded in the early postoperative period in 23 (27%) patients (table 1). The most common complication was a wound infection, which occurred in 11(13%) patients, three of whom were diagnosed as having wound infections after discharge from hospital.

**Table 1:** Post operative surgical complications

**Type of Complication**	**Number of Patients**
Wound Infection	11
Haematoma	3
Urinary Tract Infection	5
Acute Retention of Urine	2
Pain	2
Faecal Impaction	2
Pneumonia	1

Only two (2%) patients had covering colostomies at the time of sphincter repair, both of which were subsequently reversed successfully. Fifteen patients (18%) have since required further surgery: a colostomy was formed in five patients (6%), four (5%) underwent corrective surgery for scar tissue to the anal canal or vagina, four (5%) required prolapse surgery, either a Delorme's procedure or a rectopexy, and two (2%) underwent subsequent post anal repair.

Six patients (7%) were deceased at the time of the study having died from unrelated causes. Questionnaires were sent to 79 patients at the addresses known to the hospital. Overall the response rate was 59% (47/79). This group that responded did not include any male patients. The non-responders were similar in age (median age 51 years) to the responders (46 years) and the referral pattern was also similar (tertiary referrals rates 66% vs 60% respectively).

Early results revealed that 45 (96%) responders indicated that their symptoms were initially improved following surgery. When we assessed the long term outcome (≥ 5 years, range 5–12 years) of ASR, patients scored symptom improvement into one of four categories (table 2). Twenty-eight (60%) responders followed up long term still reported improvement of symptoms. No responders felt that their symptoms were worse following surgery.

**Table 2:** Symptom improvement following anterior sphincter repair

**Symptom Change Following Surgery**	**Number of Patients (% percentage)**
Improved	28 (60)
Initially Improved, but since deteriorated	17 (36)
Same	2 (4)
Worse	0 (0)

There was a significant improvement in the median linear patient assessment score comparing pre-operative with post-operative scores (3.5 (range 1–8) vs 7.0 (range 1–10) respectively, *P *< 0.001). The median St Mark's continence scores were significantly improved following surgery (14.0 (range 4–21) vs 11.0 (range 0–24) respectively, p = 0.004). Although two patients ranked themselves as totally continent (10/10) on the linear patient assessment score, they still scored 8 and 12 on the St Mark's continence score. Only one patient scored zero on the St Mark's continence score post surgery, their linear patient score was 9/10.

In addition, there was significant improvement in quality of life scores post-operatively measured by the Manchester Health Questionnaire (*P *< 0.001). Improvement in the St Mark's score and the linear assessment correlated with patient reported outcome score (Spearman 0.427, p = 0.01). Three patients who reported improvement following surgery had deterioration of continence scores, and one patient who felt that their symptoms had not changed following surgery had improvement of continence scores.

Patients were asked if they would recommend an ASR to a friend. Twenty-five (54%) responders would recommend it to a friend, 16(35%) would maybe recommend it, and 5(11%) would not recommend surgery. All the responders who had improved following surgery would recommend ASR to a friend.

Of those patients who returned completed questionnaires 37 (79%) reported obstetric injuries. Twenty-two (61%) of these reported long-term improvement following ASR as compared to 60% of all responders (p = 0.472). There was no significant difference between obstetric and non-obstetric patients with regard to anorectal physiology investigations, age at time of surgery and duration of symptoms of faecal incontinence.

Factors predictive of a long term success were analysed. No association was found between; age at time of surgery (p = 0.42), length of presenting symptoms (p = 0.518), or the length of time since surgery (p = 0.397). No predictive association was found with preoperative continence scoring systems and clinical outcome (p = 0.741).

Anorectal physiology tests had no predictive value; resting pressure (p = 0.734), squeeze pressure (p = 0.659) and rectal sensitivity (p > 0.198). There was no predictive correlation in surgical outcome between responders who had a pudendal neuropathy (unilateral or bilateral) and those who had normal PNTML measurements (p = 0.784).

Thirteen women did not undergo endoanal ultrasound as it was only introduced to the unit in 1996. Of 34 women in whom endoanal ultrasound was performed, 13 (38%) patients had defects in the external anal sphincter, 5 (15%) in the internal anal sphincter and 12 (35%) in both sphincters. Four (12%) patients had intact, but thin sphincters. There was no difference in outcome following surgery since the introduction of endoanal ultrasound (p = 0.76). Patients undergoing sphincter repair had an improved outcome if they had a defect in both sphincters, as opposed to just an external sphincter defect (p = 0.001), though the numbers are small (n = 12 and n = 13 respectively).

Three patients (6%) who returned the questionnaires had undergone previous anterior sphincter repair at other hospitals. Two patients from this group reported continued improvement in symptoms and though one initially improved, her clinical outcome has since deteriorated.

Twelve patients (14%) had repeat manometry following the ASR and of these 80% had reported improvement in symptoms. There was no difference between MRP or MSP pre- and post-surgery (50.6 ± 25 cm H_2_O vs. 46.8 ± 26.2 cm H_2_O, p = 0.247 and 35.3 ± 23 cm H_2_O vs. 35.8 ± 36 cm H_2_O, p = 0.960 respectively).

## Discussion and conclusion

Anal sphincter defects are a common cause of faecal incontinence, with obstetric injuries in female patients accounting for the majority [15]. The results from previous studies [4,5] show that early postoperative success is achieved in 70–90% of cases. Longer term results have showed lower success rates [2,11]. It remains unclear why we see a time related deterioration in clinical outcome after an initially successful sphincteroplasty. The true extent of the injury, as detected by endoanal ultrasound and more recently magnetic resonance imaging, may not be appreciated at the time of sphincteroplasty. Occult co-existent anatomical defects of the pelvic floor may contribute to poor long term outcome [16,17]. Ageing, with resulting atrophy of striated muscle, may also play a part [18]. Furthermore overzealous repairs may actually denervate or devascularise the EAS with subsequent poor outcome.

The questionnaire response rate for the study is disappointing. We report on a single surgeon's tertiary referral practice from three regions. It should be recognised that on re-contacting patients, following discharge from our care, their correspondence addresses may have changed. We can only speculate as to whether the non-responders had positive or negative results from their surgery.

In this study, 96% of patients reported an excellent initial improvement in symptoms. Of the 47 patients assessed long term (≥ 5 years), 28 (60%) maintained a good outcome. All patients underwent a standardised surgical repair and there was no difference between any subgroups of patients, yet for 40% of patients a long term successful outcome was not achieved.

There is no correlation between preoperative anorectal physiology investigations and successful outcome. However, in the five patients who went on to have colostomies fashioned following failed ASR surgery, a trend towards lower preoperative resting and voluntary contraction pressures was noted.

Previous studies looking at anorectal manometry investigations have reported conflicting results [19,20] following anterior sphincter repairs, however most studies report improvement in manometry in successful patients. Interestingly postoperative anorectal physiology in our patients revealed no improvement in resting or voluntary contraction pressures. Repairing the sphincter defect does not appear to alter the function of the striated EAS. Surgery may however have a stenosing effect and alter the compliance and length of the anal canal thus contributing to improved anal continence. Patients undergoing sphincter repair had an improved outcome success if they had a defect in both sphincters, internal and external as opposed to just an external sphincter defect (p = 0.001), the numbers however are small.

The St Mark's scores suggested that complete continence, and therefore cure, was not being achieved, however significant improvements in quality of life were demonstrated.

In this study group 32% of patients had unilateral or bilateral pudendal neuropathy diagnosed by PNTML measurement. This incidence is similar to that of a larger unselected group of 2067 patients previously published by our group [21].

Our study, like others [22] has shown that the long term outcome of sphincteroplasty does not appear to be affected by pudendal neuropathy determined by PNTML measurement. In light of this we propose that sphincteroplasty should be performed for sphincter defects despite the presence of pudendal neuropathy.

Changes in shape, length or distensibility of the sphincter may be occurring. At our unit a levatoroplasty is routinely performed. Anal sphincter repair and levatoroplasty may promote the formation of scar tissue promoting a more rigid, less distensible anal canal. These changes may be responsible for the improvement in symptoms reported.

Anterior sphincter repair with levatoroplasty is successful in improving symptoms of faecal incontinence. Even if all the non-responders had a poor result then for one third of patients this procedure remains worthwhile in the long term.

## Abbreviations

ASR Anterior Sphincter Repair

EAS External Anal Sphincter

PNTML Pudendal Nerve Terminal Motor Latency

MRP Maximal Resting Pressure

MVC Maximal Voluntary Contraction

MSP Maximal Squeeze Pressure

## Competing interests

The author(s) declare that they have no competing interests.

## Authors' contributions

BG collected the retrospective data and identified patients suitable for entry into the study. Subsequently BG compiled the manuscript draft and subsequent revisions. KT and RS were involved in the further follow up and retesting of patients (repeat anorectal physiology) as well as taking key roles in data interpretation and the formulation of the manuscript and its revisions. EK was clinical lead and the main surgeon in all of the cases, whilst also providing expert review of the scripts. All authors were members of the surgical team at various points throughout the period of study. All authors read and approved the final manuscript.

## Pre-publication history

The pre-publication history for this paper can be accessed here:



## References

[B1] Jorge JM, Wexner SD (1993). Etiology and management of fecal incontinence. Diseases of the Colon and Rectum.

[B2] Halverson AL, Hull TL (2002). Long-term outcome of overlapping anal sphincter repair. Diseases of the Colon and Rectum.

[B3] Sultan AH, Kamm MA, Hudson CN, Bartram CI (1994). Third degree obstetric anal sphincter tears: Risk factors and outcome of primary repair. BMJ.

[B4] Engel AF, Kamm MA, Sultan AH, Bartram CI, Nicholls RJ (1994). Anterior anal sphincter repair in patients with obstetric trauma. British Journal of Surgery.

[B5] Fang DT, Nivatvongs S, Vermeulen FD, Herman FN, Goldberg SM, Rothenberger DA (1984). Overlapping sphincteroplasty for acquired anal incontinence. Diseases of the Colon and Rectum.

[B6] Snooks SJ, Henry MM, Swash M (1985). Faecal incontinence due to external anal sphincter division in childbirth is associated with damage to the innervation of the pelvic floor: a double pathology. Br J Obstet Gynecol.

[B7] Gilliland R, Altomare D, Moreria H, Oliveira L, Gilliland J, Wexner S (2003). Pudendal neuropathy is predictive of failure following anterior overlapping sphincteroplasty. Diseases of the Colon and Rectum.

[B8] Londono-Schimmer EE, Garcia-Duperly R, Nicholls RJ, Ritchie JK, Hawley PR, Thompson JP (1994). Overlapping anal sphincter repair for faecal incontinence due to sphincter trauma: Five year follow-up functional results. International Journal of Colorectal Disease.

[B9] Nikiteas N, Korsgen S, Kumar D, Keighley MR (1996). Audit of sphincter repair. Factors associated with poor outcome. Diseases of the Colon and Rectum.

[B10] Chen AS, Luchtefeld MA, Senagore AJ, Mackeigan JM, Hoyt C (1998). Pudendal nerve latency. Does it predict outcome of anal sphincter repair?. Diseases of the Colon and Rectum.

[B11] Malouf AJ, Norton CS, Engel AF, Nicholls RJ, Kamm MA (2000). Long-term results of overlapping anterior anal-sphincter repair for obstetric trauma. Lancet.

[B12] Young CJ, Mathur MN, Eyers AA, Solomon MJ (1998). Successful overlapping anal sphincter repair: Relationship to patient age, neuropathy and colostomy formation. Diseases of the Colon and Rectum.

[B13] Vaizey CJ, Carapeti E, Cahill JA, Kamm MA (1999). Prospective comparison of faecal incontinence grading systems. Gut.

[B14] Bug GJ, Kiff ES, Hosker G (2001). A new condition-specific health-related quality of life questionnaire for the assessment of women with anal incontinence. Br J Obstet Gynecol.

[B15] De Leeuw JW, Vierhout ME, Stuijk PC, Audwerda HJ, Bac DJ, Wallenburgh HCS (2002). Anal sphincter damage after vaginal delivery: Relationship of anal endosonography and manometry to anorectal complaints. Diseases of the Colon and Rectum.

[B16] Fletcher JG, Busse RF, Riederer SJ, Hough D, Gluecker T, Harper CM, Bharucha AE (2003). Magnetic resonance imaging of anatomic and dynamic defects of the pelvic floor in defecatory disorders. The American Journal of Gastroenterology.

[B17] Rociu E, Stoker J, Eijkemans MJC, Schouten WR, Lameris JS (1999). Fecal incontinence: Endoanal US versus endoanal MR imaging. Radiology.

[B18] Barrett JA, Brocklehurst JC, Kiff ES, Ferguson G, Faragher EB (1989). Anal function in geriatric patients with faecal incontinence. Gut.

[B19] Oliveira L, Pfeifer J, Wexner SD (1996). Physiological and clinical outcome of anterior sphincteroplasty. British Journal of Surgery.

[B20] Felt-Bersma RJF, Cuesta MA, Koorevaar M (1996). Anal sphincter repair improves anorectal function and endoscopic image: A prospective clinical study. Diseases of the Colon and Rectum.

[B21] Hill J, Hosker G, Kiff ES (2002). Pudendal nerve terminal motor latency measurements: What they do and do not tell us. British Journal of Surgery.

[B22] Tjandra J, Han W, Ooi B, Thorne M (2001). Unilaterally prolonged pudendal nerve terminal motor latency does not contraindicate repair of sphincter defects. Diseases of the Colon and Rectum.

